# *Actinidia arguta* Leaf as a Donor of Potentially Healthful Bioactive Compounds: Implications of Cultivar, Time of Sampling and Soil N Level

**DOI:** 10.3390/molecules26133871

**Published:** 2021-06-24

**Authors:** Jan Stefaniak, Barbara Łata

**Affiliations:** Laboratory of Basic Sciences in Horticulture, Department of Plant Protection, Institute of Horticultural Sciences, Warsaw University of Life Sciences—SGGW, Nowoursynowska 159, 02-776 Warsaw, Poland; jstefaniak@gmail.com

**Keywords:** kiwiberry, glutathione-ascorbate redox state, phenolics, antioxidant activity

## Abstract

The aim of this study was to assess the enzymatic and non-enzymatic antioxidant status of kiwiberry (*Actinidia arguta*) leaf under different N regimes tested three times in field conditions during the 2015 growing season in two cultivars (‘Weiki’ and ‘Geneva’). Leaf total antioxidant capacity using ABTS, DPPH and FRAP tests was evaluated in the years 2015 to 2017, which experienced different weather conditions. Both cultivars exhibited a significant fall in leaf L-ascorbic acid (L-AA) and reduced glutathione (GSH) as well as global content of these compounds during the growing season, while total phenolic contents slightly (‘Weiki’) or significantly (‘Geneva’) increased. There was a large fluctuation in antioxidative enzyme activity during the season. The correlation between individual antioxidants and trolox equivalent antioxidant capacity (TEAC) depended on the plant development phase. The study revealed two peaks of an increase in TEAC at the start and end of the growing season. Leaf L-AA, global phenolics, APX, CAT and TEAC depended on the N level, but thiol compounds were not affected. Over the three years, TEAC decreased as soil N fertility increased, and the strength of the N effect was year dependent. The relationship between leaf N content and ABTS and FRAP tests was highly negative. The antioxidant properties of kiwiberry leaves were found to be closely related to the plant development phase and affected by soil N fertility.

## 1. Introduction

Reactive oxygen species (ROS) and antioxidants are inherent elements in the metabolism of aerobes. ROS are produced during normal metabolic processes and serve as cell-signalling molecules [1]. If their concentration is too high, ROS can cause severe damage to biological molecules, especially DNA, lipids and cellular proteins [2,3]. In turn, enzymatic and non-enzymatic antioxidants form a scavenging repair system that tries to prevent ROS from reaching harmful levels or deals with molecules that have been oxidatively damaged [2]. Many civilisation diseases are caused by the human body being exposed to oxidative stress [4,5,6]. Research has demonstrated that nutrition plays a very important role in the prevention of chronic diseases [5,7]. To maintain good health, humans are highly encouraged to consume fruits and vegetables [8], thus there is a constant large demand for fresh health-promoting food. Functional food not only provides a nutritional function but also contributes to the reduction of risk factors of several diseases or enhances certain physiological functions in the body [9]. Therefore, new natural sources of high antioxidant activity, which could be classified as functional food, are constantly under scientific consideration [5]. *Actinidia arguta* (Siebold et Zucc.) Planch. ex Miq.), commonly known as kiwiberry, is a new product on the market that is experiencing growing consumer acceptance as well as an increasing area of production worldwide [10]. Kiwiberry, a fruit with an edible skin, is characterised by high contents of phenolics, carotenoids, vitamin C and thiol compounds [11,12] and it exhibits strong antioxidant activities in DPPH, FRAP and ABTS tests [12,13,14,15]. These fruit traits provide a basis for considering kiwiberry as a health-promoting product (functional food) [14,16]. In fact, both the fruits and leaves of *A. arguta* have also been evaluated in terms of biological activity [17,18,19]. They have revealed high antioxidant efficiency in DPPH and FRAP tests and demonstrated the ability to protect biological membranes from multiple reactive oxygen and nitrogen species due to being a rich source of bioactive, especially polyphenolic, substances. *A. arguta* leaves are proposed as a potentially valuable food additive that offers protection against many diseases caused by oxidative stress [17,19]. High antioxidant potential is derived from the specific role of leaves in plant metabolism, and generally leaves contain higher concentrations of bioactive compounds and related enzymes than fruit [18,20,21].

Plant antioxidant metabolism is genetically determined [15,22,23,24,25], but environmentally modified due to several stresses and also agronomic approaches [26,27,28]. To obtain a high-quality product, these interrelationships need to be studied in detail. Recent research undertaken on kiwiberry provides valuable information on the potential leaf quali-quantitative distribution pattern of phenolics and leaf biological activity, however, no consideration has been given to cultivar differences or other factors [17,19]. There are numerous reports of pre-harvest and post-harvest factors affecting plant bioactive compounds and plant tissue biological activity, which in effect can lead to a different quality of the final bioproducts [8,26,29,30,31,32]. It would appear that all these factors should be of special interest since the leaves of fruit plants are not a food item or food additive in the classic understanding of these, and factors affecting leaf quality should be acknowledged in detail. For many years, special attention has been paid to N management due to its great impact on food quality and the environment [33,34]. The relationship between leaf antioxidant metabolism and soil essential nutrients, including N, has recently been discussed in some papers, indicating a negative, neutral or positive relationship depending on the N level, nutrient type or other factors specific to the studies. For instance, N availability is reported to influence CAT and APX activities and the concentration of ascorbic acid in *Arabidopsis thaliana* L. leaves [35] and *Coffea arabica* L. leaves [26]. Appropriate N adjustment is related to the content of polyphenolic compounds and antioxidant activity status in potatoes [36]. In turn, N fertilisation has been reported to positively affect the total glutathione concentration and the proportion of its reduced form in *Vaccinium myrtillus* L. [37]. In summary, the high quality and functionality of food products, along with sustainable production and possible re-use of by-products, are modern goals of manufacturers and researchers of food products [19,38]. To embrace the concept of sustainability and waste reduction [38], optimal N fertilisation in food production should be targeted in order to reduce soil nitrate losses [12,39].

Another important factor regarding tissue chemical content and composition is the plant development phase [26,30,31,40]. Time of harvest can determine possible applications of the plant material because plant chemical composition is influenced by the seasons and may vary depending on the month, leading to products with different compositions and pharmacological properties [31]. For instance, blueberry leaves are characterised by different concentrations of phenolic compounds and antioxidant activities when affected by different seasons and cultivars [41] or when affected by cultivar and time of harvest [30,40]. Harvest time also influences antioxidant enzyme activities in coffee leaves [26]. Leaf sampling is a common and efficient way to check plant nutrition status and verify fertilisation effectiveness [42]. However, leaves can also be tested to verify the overall plant condition [43] or can be considered as an extraordinary source of value-added bioactive compounds with pro-healthy potential [17,18,19]. To date no comprehensive survey of kiwiberry leaf antioxidant potential has been undertaken that includes several factors such as cultivar, time of sampling or controlled plant mineral nutrition with a special focus on N. This paper presents the results concerning kiwiberry leaf enzymatic and non-enzymatic antioxidants under different N regimes tested three times in field conditions in two cultivars during the growing season. Leaf total antioxidant capacity using ABTS, DPPH and FRAP tests was evaluated over three years in differing weather conditions in 2015, 2016 and 2017. It is hoped that these results offer a good estimation of leaf tissue status as a potential donor of bioactive compounds and the extent to which some factors can change this status. It supplements and contributes to current knowledge about *Actinidia arguta* plants as a comprehensive source of food rich in bioactive compounds.

## 2. Results and Discussion

A large amount of recent research has indicated leaves to be an extraordinary source of value-added compounds [20,22,24,41,44,45], including some studies on kiwiberry leaves (*Actinidia arguta*) [17,18,19]. Such material is proposed for use in the food, pharmaceutical and cosmetic industries [19,20,44]. Apart from the obvious influence of genetic factors, edaphoclimatic conditions, agronomic approaches and plant development phase are important for the quality of potential raw material. There is little in the literature about the influence of these factors in the context of fruit plant leaves [21,26,40,41] and nothing concerning *Actinidia arguta* leaves.

### 2.1. A. arguta Leaf Antioxidant Properties as a Function of Cultivar and Harvest Time

Plant antioxidant status provides important information about the plant metabolism in given environmental conditions [35,46,47]. Another important consideration is the plant-derived food harvest time corresponding with its high biological value since the antioxidant metabolism fluctuates due to plant growth and development as well as biotic or abiotic stress factors [21,26,40,41]. There are no data about the antioxidant metabolism in kiwiberry leaves. However, in the last few years, the fluctuation in leaf essential nutrients over growing seasons has been a topic of discussion [42,48]. Since *A. arguta* is a dioecious woody vine, leaf nutrient concentration varies not only by year or sampling date but also by plant gender and shoot type (determinate and indeterminate) for many tested nutrients [48]. Therefore, in an assessment of kiwiberry leaf antioxidant capacity, all these factors should be precisely defined since they can influence the final product.

**Figure 1:**
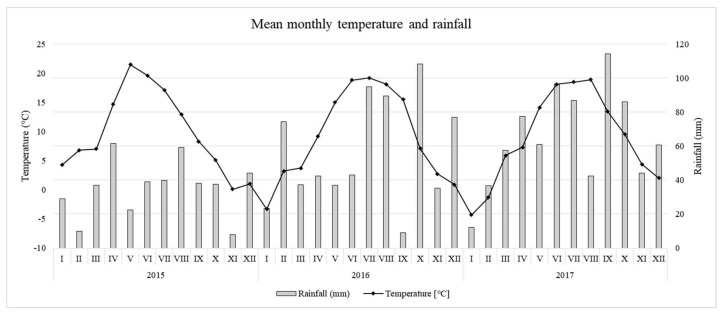
Data on average temperatures and rainfall at the experimental site in the 2015, 2016 and 2017 growing period. Long-term averages calculated for each month (I–XII) in the period 1982–2012 were −4.3, −3, 1.9, 8.2, 13.1, 16.5, 17.9, 17.4, 13.4, 8.7, 3, −1.6 °C and 25, 24, 28, 35, 57, 72, 75, 62, 44, 36, 39, 34 mm for temperature and rainfall respectively. Average temperature and rainfall for the period 1982–2012 were 7.6 °C and 44.25 mm respectively.

The weather data collected throughout the experiment are presented in Figure 1. As can be seen, the weather conditions during the experiment differed in terms of temperature and precipitation, which will be commented on in more detail in the following sections. Statistical analysis revealed the great impact of leaf sampling time on its antioxidant properties (Table 1). Leaf sampling time effect has been statistically proved for most of the tested antioxidants at a very high probability level (α = 0.001), especially for the ‘Geneva’, where the F value frequently exceeded 60 (Table 1). The most significant effect of leaf sampling time was in the case of total ascorbate content (‘Weiki’, F value = 103) and L-AA (‘Geneva’, F value = 151). Based on the F value, the leaf sampling time displayed a more comparable influence on ‘Geneva’ leaf antioxidant properties as compared to ‘Weiki’. In contrast to leaf sampling time, the N level showed much less importance regarding examined antioxidants. However, the N effect was statistically proved in the case of L-AA, APX and CAT activity for both cultivars and at a similar probability level (α in the range 0.01–0.001). 

**Figure 2:**
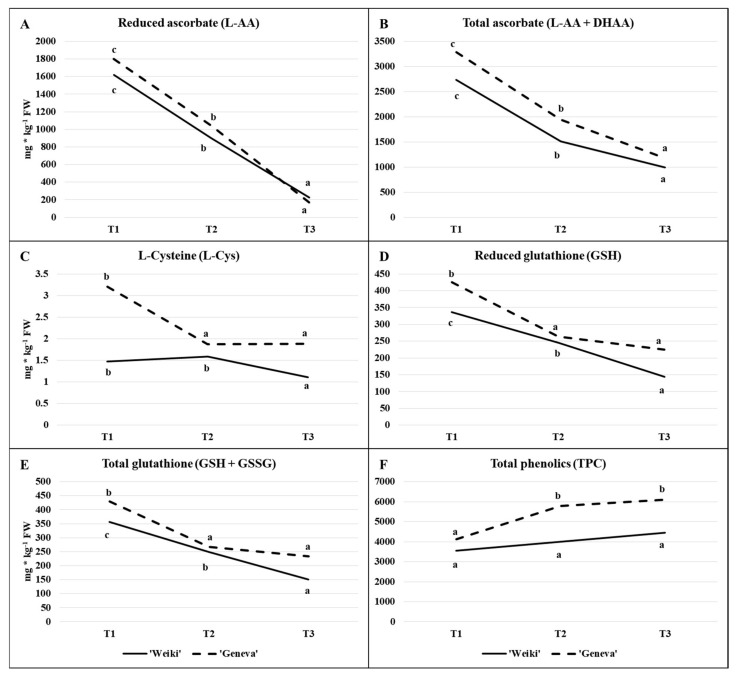
Concentration of bioactive compounds (mg kg^−1^ FW) of: (**A**) reduced ascorbate, (**B**) total ascorbate, (**C**) L-cysteine, (**D**) reduced glutathione, (**E**) total glutathione, (**F**) total phenolics; depending on time of sampling in the leaves of two *Actinidia arguta* cultivars in 2015. Presented data are means for three soil N levels and three replications. Values marked with a different letter on each chart differ significantly at *p* ≤ 0.05 (Tukey HSD test). Cultivars were analysed separately; T1—(28.05.15) the stage of fully developed leaves, T2—(16.07.15) the peak of the most intensive fruit growth and T3—(27.08.15) the beginning of fruit physiological maturity.

**Figure 3:**
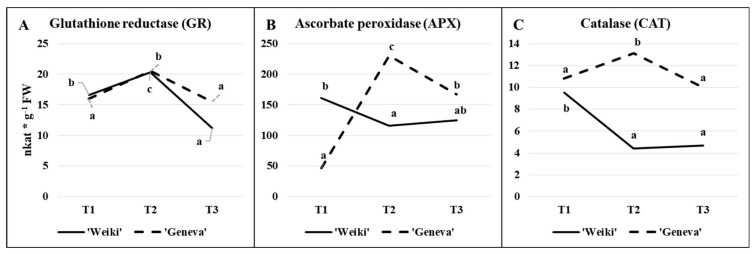
Antioxidative enzyme activity (nkat g^−1^ FW) of: (**A**) glutathione reductase, (**B**) ascorbate peroxidase, (**C**) catalase; depending on the time of sampling in leaves of two *A. arguta* cultivars in 2015. Presented data are means for three soil N levels and three replications. Values marked with a different letter in each group differ significantly at *p* ≤ 0.05 (Tukey HSD test). Cultivars were analysed separately; T1—(28.05.15) the stage of fully developed leaves, T2—(16.07.15) the peak of the most intensive fruit growth and T3—(27.08.15) the beginning of fruit physiological maturity.

In detail, the time-dependent changes of the ascorbate and glutathione status, the concentration of L-cysteine (the precursor to glutathione) and global phenolics show Figure 2, while Figure 3 highlights the activity of selected antioxidant enzymes (GR, APX and CAT) in leaves collected from terminating shoots of female plants. Two cultivars were monitored to identify potential differences. The above-mentioned ROS-scavenging enzymes and non-enzymatic antioxidants are crucial for ROS homeostasis in plants [47,49]. Overproduction of ROS is caused by stress conditions and the increased production of ROS during stresses is also thought to act as a signal for the activation of stress response pathways [50]. Antioxidants also undergo remarkable changes during plant growth and development [46]. In this study, irrespective of cultivar, leaf sampling time significantly influenced the tested antioxidant concentrations and antioxidative enzyme activity (Figure 2; Figure 3, Table 1).

A strong decrease in L-AA and total ascorbate (L-AA + DHAA) concentrations progressed with each time point at a very similar intensity for both cultivars (Figure 2A,B). Similar to ascorbate, leaf GSH and total glutathione concentrations (GSH+GSSG) also decreased at each harvest time with some variation depending on the cultivar (Figure 2D,E). The glutathione concentration decreased significantly between T1 and T2 in ‘Geneva’ leaves, then remained similar at T3. In ‘Weiki’ leaves, the concentration of this compound decreased steadily until the last leaf sampling. These cultivar differences can partly be explained by the time-dependent changes in the content of L-Cys (Figure 2C), a precursor to glutathione. On average, L-Cys content was higher in ‘Geneva’ leaves, especially at the start of the growing season (T1), and remained at the same level at the T2–T3 measured points. In ‘Weiki’, there was a significant decrease in L-cysteine concentration between the T2 and T3 measurements. A higher and more stable L-Cys level from the middle to end of the season in ‘Geneva’ might have generated these differences in the glutathione content and fluctuation over the tested time points.

Looking at the changes in enzyme activity (Figure 3A–C), large fluctuations can be observed over the period tested. Time-dependent changes in the activity of GR (Figure 3A), an enzyme involved in the regeneration of GSSG to GSH, were greater in ‘Weiki’ than the ‘Geneva’ cultivar. Taking into account L-Cys content and changes and GR activity, it seemed the ‘Geneva’ leaf glutathione regeneration system was more efficient than that of ‘Weiki’. However, the picture of the time-dependent changes in GR activity was quite similar in both cultivars. In turn, time-dependent changes of APX (Figure 3B) and CAT (Figure 3C) differed significantly in the tested cultivars. The APX and CAT activity in ‘Geneva’ leaves followed a similar pattern to GR activity, increasing considerably at T2 then decreasing at T3. In ‘Weiki’, however, an initial drop in APX and CAT activity was observed in the middle of the growing season and remained relatively stable until the last harvesting time point. Ascorbate-glutathione cycle and related enzymes are involved, inter alia, in the removal of hydrogen peroxide, one of the intermediate products of the reduction of molecular oxygen [47,51]. Dynamic changes in the components building this system might be related to the aging process and to the response to changing environmental factors. In 2015, high temperatures in May, June and July were accompanied by low rainfall compared with long-term averages, while in August there was a large drop in temperature and an increase in rainfall (Figure 1). This course of weather might also have affected the antioxidant metabolism. Seasonal changes in ascorbate–glutathione status and antioxidative enzyme activity have been studied in relation to temperature and/or light intensity or as a function of acclimation strategy [52,53]. Regarding the plant development stage, Kandlbinder et al. [35] report differences in CAT and APX activity between young and older leaves of *Arabidopsis thaliana*, while in coffee leaves a high activity of the enzymatic antioxidant system during the coffee fruit ripening stage was noted [26]. In the present study, enzyme activity and ascorbate–glutathione contents were only tested at three time points throughout the growing season, which is probably why there is a large variation between tested sampling times. To the best of the authors’ knowledge, the glutathione–ascorbate cycle in *A. arguta* leaves and other fruit plant leaves is not presented in the literature.

Much more research has been devoted to leaf phenolic content, both total concentration and individual compounds, and total antioxidant activity [18,20,22,24,44,45], but these have rarely been studied in relation to different harvest times [41]. Phenolic compounds are of great interest for human nutrition and plant health, with reports of several beneficial properties in [7,8,46]. In contrast to ascorbate and glutathione, the total phenolic content significantly (‘Geneva’) or slightly (‘Weiki’) increased throughout the growing season (Figure 2F). Considerably lower amounts of phenolics were found in multiple cultivars of *Vaccinium ashei* leaves sampled at the end of fruit ripening (December—late spring/early summer in Brazil) compared with those sampled in March (late summer/early autumn) [41]. However, in relation to the individual compounds separated by the HPLC method in this study [41], such as chlorogenic acid or rutin, which dominated in the *Vaccinium ashei* leaf extract, the directions of changes were not as uniform between different cultivars. Therefore these changes may be dependent on the quali-quantitative leaf phenolic pattern of a given species or even cultivars. Quantitative and qualitative variations in phenolic content between species and cultivars are common phenomena [11,15,54]. In turn, in leaves of lingonberry, the sum of phenolic compounds appeared stable from May to September 2013, while it significantly increased from July to September 2014, outlining a major annual effect during the growth season. Interestingly, seasonal variations also differ at the phenolic class level [21]. During tissue differentiation and organ development, the phenolic profiles often undergo remarkable changes, indicating the integration of their metabolism in programmes of growth and development [46].

Summarising this part of the results, it can be concluded that the ascorbate–glutathione cycle and related enzymes show large changes in concentration/activity, indicating a close relationship with the plant development cycle and high sensitivity to overall external conditions. Leaf global phenolic variation was rather low and cultivar dependent: in one cultivar, phenolic concentration did not change over the period tested and in the other, it significantly increased. However, it can be expected that the content of some individual phenolics may increase and others decrease [21,30,46]. This should be clarified in subsequent studies since the antioxidant properties of individual phenolic compounds differ [25].

The correlation between individual compounds and total antioxidant capacity as well as between leaf N contents and TEAC measured in different tests over three points of time in 2015 is presented in Table 2. The correlation significance depended on leaf sampling time but was positive for most antioxidant components in the ABTS and FRAP assays. This relationship has frequently been reported [18,19,22,24]. Surprisingly, DPPH was not correlated or exhibited a negative relationship with the examined compounds, which has rarely been presented by other studies [41]. Some interesting findings regarding the presented positive correlations (ABTS and FRAP vs. tested antioxidants) over the growing season should be noted. At the last time point, there was no correlation between antioxidant capacity and ascorbate content, whereas in the case of glutathione and antioxidant capacity this relationship was strengthened, although both components decreased considerably over the season. Such differences might be related to enzyme activities. At the end of the season, there were still positive correlations between GR and FRAP or ABTS tests, but not for APX, which is related more to ascorbate regeneration. Such findings indirectly confirm the dynamics and involvement of these compounds in the plant development stages and reception of environmental conditions. The correlation coefficient between antioxidant capacity and phenolics remained relatively similar at each measurement point, which is in line with moderate changes in these compounds over the studied period. 

In turn, the relationship between leaf N content and TEAC measured using ABTS and FRAP assays was highly negative, especially at T2 point time. The opposite relationship occurred in the case of DPPH, namely the found correlation was positive (Table 2).

**Figure 4:**
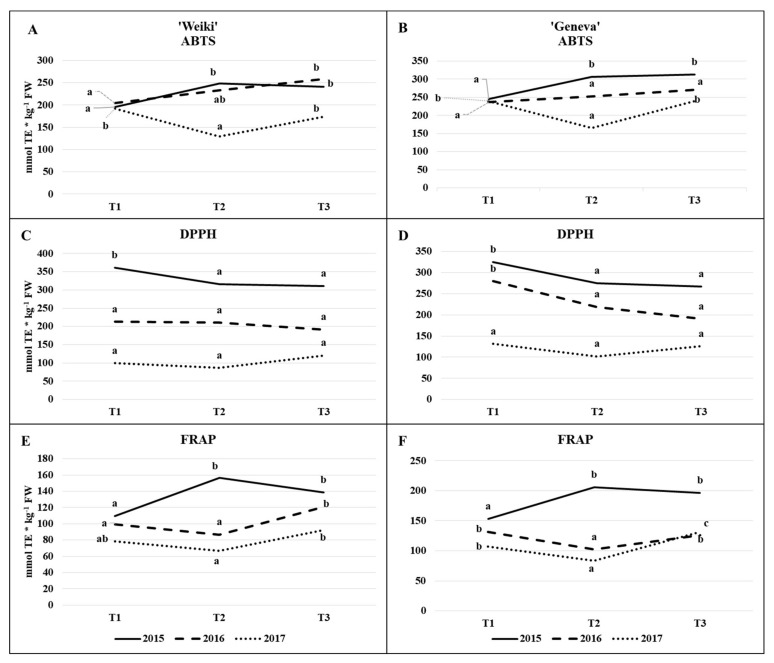
Total antioxidant capacity measured in ABTS ((**A**) ‘Weiki’, (**B**) ‘Geneva’), DPPH ((**C**) ‘Weiki’, (**D**) ‘Geneva’) and FRAP ((**E**) ‘Weiki’, (**F**) ‘Geneva’) tests (mmol TE kg^−1^ FW; TE—trolox equivalents) depending on the time of sampling in different growing seasons (2015, 2016 and 2017) in *A. arguta* leaves. Presented data are means for three soil N levels and three replications. The subsequent years and cultivars were analysed separately; T1—(28.05.15/2.06.16/8.06.17) the stage of fully developed leaves, T2—(16.07.15/14.07.16/18.07.17) the peak of the most intensive fruit growth, T3—(27.08.15/25.08.16/30.08.17) the beginning of fruit physiological maturity. ABTS: 2,2′-azino-bis(3-ethylbenzothiazoline-6-sulphonic acid); DPPH: 2,2-diphenyl-1-picrylhydrazyl; FRAP: ferric reducing antioxidant power.

In view of the above results, research on antioxidant capacity continued in order to assess leaf biological value fluctuations based on ABTS, FRAP and DPPH tests, using the same cultivars as in the case of individual compounds and maintaining the same soil conditions in 2016–2017 (Table 3, Figure 4A–F). The influence of both principal factors (leaf sampling time and N level) on TEAC, has been explicitly confirmed based on FRAP followed by ABTS assay (Table 3). ‘Geneva’ was more responsive in terms of N feeding than ‘Weiki’ since in all examined growing seasons, both in the ABTS and FRAP tests, the effect of soil N fertility on TEAC was proven. In contrast to the FRAP and ABTS tests, the effect of time or nitrogen level on TEAC has not been convincingly proven using the DPPH test (Table 3).

The interaction between the sampling time and N level did not in principle appear (Table 3). The year effect was clearly marked also.

High variability in consecutive years in the distribution of precipitation and temperature over the growing season, caused by climate change among other things, led to significant differences in the results of the open field study from year to year [14,55]. Irrespective of the cultivars and tests used, TEAC was considerably higher in 2015 than in 2016 and 2017 (Figure 4A–F). The year 2015 differed considerably from the subsequent two years in terms of temperature and rainfall, as underlined at the start of this section (Figure 1). Two of the tests used (ABTS and FRAP, Figure 4A,B,E,F) showed a significant increase in leaf antioxidant capacity from the end of May (T1) to mid-July (T2) in 2015. This may have been a response to drought stress or light intensity. Changes in the capacity of the antioxidant apparatus due to abiotic stresses have frequently been described [32,35,36]. In contrast to the tests described above, DPPH decreased significantly in the corresponding period of time. This may partly be explained by FRAP and ABTS being positively correlated with almost all the individual antioxidants tested (Table 2), while DPPH showed a predominantly negative correlation in the experiment discussed. Excluding 2015 for its considerable differences in terms of weather, and taking into account the FRAP and ABTS tests that best correlated with individual antioxidants in this study, the highest TEAC capacity was recorded at the stage of young fully-developed leaves (T1) and at the start of fruit physiological maturity (T3). This reflects the time-dependent changes in the content of individual compounds evaluated in 2015 (Figure 2A–F). At T1, leaves were characterised by a high content of thiol compounds and ascorbate, and the lowest concentration of phenolic compounds. At T3, the phenolic concentration increased, which probably compensated for the decrease in the remaining tested antioxidants. Maintaining a high antioxidant capacity in these two periods may also indicate that they are of great importance to kiwiberry plant development. Reis et al. [26] indicate that the antioxidant system is very active during the coffee fruit-ripening stage.

Compared with FRAP and ABTS, the DPPH value (Figure 4C,D) was relatively stable over the 2016–2017 growing seasons, especially for the ‘Weiki’ cultivar. Irrespective of the assay used, no major differences were found in the pattern of time-dependent changes in antioxidant capacity between cultivars.

### 2.2. Relationship between Soil N Level and Leaf Chemical Composition as Well as Antioxidant Capacity

Nutrient availability is a key controlling factor of biomass production and yield quality, but there are also increasing expectations in society for the development of an ecologically friendly management of mineral nutrition in agro-ecosystems [56]. Although it is essential for high crop yields, the abundant use of nitrogen causes substantial risks to the climate, human health and ecosystems [33,34]. Therefore an assessment of the relationship between N dose and the yield attributes is crucial.

Presented data are means for the three sampling times and three replications (n = 9 ± SD) obtained in 2015. Values marked with a different letter in rows differ significantly at *p* ≤ 0.05 (Tukey test). N1, N2, N3—30, 50 and 80 mg N kg^−1^ DW respectively; DHAA: dehydroascorbic acid. In this study, the N effect on the tested antioxidants was several times lower than the time of leaf sampling, and concerned only selected components (Table 1). The distinct adverse effect of increasing N soil supply on the content of L-AA was observed in the leaves of both cultivars (Table 4). The N2 level was enough to decrease the L-AA concentration in ‘Geneva’ leaves compared with the N1 treatment, while L-AA concentration in ‘Weiki’ leaves decreased with the highest N treatment (N3). However, the total ascorbate pool remained at a similar level regardless of soil N fertility. In both cultivars, thiol compounds and GR activity were not dependent on soil N level (Table 4; Table 5). In turn, the activity of other enzymes related to the ascorbate-glutathione cycle was N and cultivar dependent (Table 5). In the leaves of ‘Weiki’, the N2 treatment resulted in significantly higher APX and CAT activity compared with the lowest (N1) and highest N treatments (N3). In ‘Geneva’, plants supplied with the lowest N doses exhibited a significant increase in leaf APX and CAT activity. *Arabidopsis thaliana* leaves revealed distinct patterns of redox responses dependent on the type of nutrient deficiency [35]. Ascorbic acid contents were strongly upregulated in N-deprived leaves, while CAT and APX activities decreased significantly. CAT is described as a sensitive indicator of nutrient deficiency in the developmental gradient of leaves. The ascorbate pool remains fully reduced, whereas the glutathione pool becomes more oxidised, particularly in old leaves of N-starved plants [35]. Since ascorbate does not contain N, upregulation of ascorbate synthesis is proposed as a resource-saving mechanism to provide low molecular mass antioxidants in nutrient-deprived plants. The enzymatic activities of APX and GR increased in the leaves of all kiwifruit genotypes when subjected to higher levels of salt stress [57].

In contrast, in coffee leaves (*Coffea arabica* L.) antioxidant enzymes (CAT, APX, SOD) are more active in N-deficient plants, indicating a clear inverse relationship between antioxidant enzyme activities and applied N [26]. In this study, the highest pool of L-AA was at the lowest soil N fertility, while CAT activity was highest in leaves of ‘Geneva’ only. In turn, the glutathione pool was kept at a reduced state, probably due to the stable level of GR activity, which was not influenced by N fertilisation. The recently described kiwiberry fruit total ascorbate and glutathione pools are not dependent on soil N fertility, while enzymatic antioxidants are activated under the highest N dose [12]. As revealed in a previous study based on chlorophyll *a* fluorescence measurements, ‘Weiki’ and ‘Geneva’ express different sensitivities to nitrogen deficiency [43]. Moreover, it should be noted that tested N levels in this study were from low (N1) to high (N3), and plants were not N-deprived because a state of such deprivation is difficult to achieve in field conditions and due to the mineralisation of organic matter. A low N level can be considered as the natural content related to the type and culture of the given soil. Testing 20 lettuce genotypes (*Lactuca sativa* L.) with low N, Zhou et al. [58] observed an increase in plant concentrations of vitamin C, glutathione and phenolic compounds compared with high N conditions. However, because of reduced biomass under N limitations, not all lettuce genotypes exhibited increased antioxidant yields or total antioxidant capacity yield. Therefore, skilful N management should consider the balance between yield and quality [12]. In conclusion, the ascorbate-glutathione cycle could be related to a large number of factors such as species, plant organ and development phase, crop management and weather conditions. In open field studies, the effect of these factors on the antioxidant metabolism can be also complicated by the interaction between various factors.

The accumulation of phenolic compounds in plant tissues is often negatively affected by high N nutrition [12,32,46]. An inverse relationship between N availability and the concentration of phenolic compounds not restricted to a specific developmental stage or cultivation method has recently been reported in the leaves of cereals [32]. Plants grown under stress conditions often produce and accumulate phenolic stress metabolites. N-deficient plants suffer from light stress due to their limited photosynthetic capacity, and unfertilised plants are more stressed by an excess of light energy and need to accumulate more antioxidants to prevent photodamage [32,59]. An increase in N level as a signal for the downregulation of genes encoding for enzymes involved in the phenylpropanoid metabolism has been described for tobacco [60]. Nitrogen starvation significantly increases PAL activity in the leaf and root tissues of yarrow (*Achillea collina* Becker ex Rchb.) [61]. Regarding N-dependent changes in kiwiberry TPC in the present study, they were expressed differently by cultivar (Table 4). A clear negative impact on TPC was exhibited by leaves of ‘Geneva’, but no effect was apparent in ‘Weiki’ leaves. This, therefore, confirms the need to assess cultivar variation in terms of responses to environmental conditions [58].

This long-term study revealed an inverse relationship between increasing soil N level and antioxidant capacity (Table 6). The clearest evidence of this was observed in the ABTS and FRAP tests. Results similar to those for leaves have been obtained in other studies [58,61,62]. The addition of inorganic N leads to a significant decrease in leaf-blade FRAP in spinach (*Spinacia oleracea* L. cv. Manatee) [62]. The results of Zhou et al. [58] suggest that even though low N conditions generally tend to improve the antioxidant qualities of lettuce, the extent of this effect is highly dependent on genotype. Antioxidant capacity is also higher in the leaves and roots of N-starved yarrow compared with the control plants [61].

The recently described kiwiberry fruit antioxidants revealed that total ascorbate and glutathione pools were not dependent on soil N fertility, phenolic content or TEAC decreases, while enzymatic antioxidants were activated under the highest N dose [12]. Comparing the nitrogen-dependent metabolisms of non-enzymatic antioxidants in kiwi leaves and fruits, it can be concluded that they are quite similar. It would appear that fluctuations in antioxidative enzyme activity are more variable and are related to factors other than N level (e.g., environmental or/and genetic factors).

## 3. Materials and Methods

### 3.1. Experimental Field, Weather and Soil Conditions

Leaf samples of ‘Weiki’ and ‘Geneva’ cultivars were acquired from the authors’ scientific project partner, the commercial A. arguta orchard located in Bodzew, Mazowieckie Voivodeship, Poland (51°47′49.9″ N + 20°48′44.0″ E) in 2015, 2016 and 2017. Examined cultivars varied with respect to leaf surface (‘Weiki’ is characterized by a smaller leaf area compared to ‘Geneva’), while both cultivar show similar chlorophyll content [63]. Both cultivars expressed similar phenology with the difference that ‘Geneva’ fruits begin to ripen about 2 weeks earlier than ‘Weiki’. The experiment was set up using a randomised block design with two factors (cultivar and N dose) and three replications (four female plants for each replication). Based on the overall recommendations for orchard soils and the advised range of 25–50 mg N kg^−1^, three soil N levels were chosen for examination in this experiment: 30 (N1), 50 (N2) and 80 (N3) mg N kg^−1^ of dry soil weight (DW). These are considered to be low, medium and high supply levels respectively. Plant rows were fertilised with ammonium nitrate (34% N) at the beginning of April and again four and eight weeks later in three equal doses. The weather data were collected throughout the experiment using the Vantage Pro2 Plus weather station (Davis, CA, USA). Weather data for the 2015–2017 seasons are presented in Figure 1. More detailed descriptions concerning experiment design, soil sampling and analysis, and mineral plant nutrition can be accessed in recently published works [12,42].

### 3.2. Leaf Sample Collection and Analysis

Leaf samples for antioxidant analysis were collected at three time points during the growing season, starting on 28 May in 2015, 2 June in 2016 and 8 June in 2017, and continuing every six weeks until the end of August (T1–T3 respectively). T1 represented the stage of fully developed leaves, T2 the peak of the most intensive fruit growth, and T3 the start of fruit physiological maturity (seed colour changed from white to brown). At each time point, three leaf samples per treatment were collected. Each sample consisted of 20 fully developed leaves collected from terminating shoots of female plants, located at the same height of the canopy. Terminating shoots end their growth quickly, so it can be assumed that in the following time point physiologically older leaves were sampled. Leaves of a similar size (for a given cultivar) were gathered evenly from shoots on both sides of each plant and represented the typical appearance for the particular N treatment. Leaves were frozen in liquid nitrogen and transferred to a freezer at −80 °C. The leaf samples were ground in mortars with liquid nitrogen to a fine powder before analysis.

### 3.3. Bioactive Compound Measurements

Thiol compounds and ascorbate. The concentration of ascorbate, glutathione and L-cysteine compounds was determined using high-performance liquid chromatography (HPLC) [29]. First, 50 mg and 100 mg of leaf tissue powder were taken to determine thiol compounds and ascorbate compounds respectively. The powder was suspended in 0.1 M HCl containing PVPP, and centrifuged for 20 min at 4 °C at 21,900× *g* or 48,000× *g* for determination of glutathione and ascorbate respectively. Total contents of glutathione (tGSH: GSH + GSSG) and L-cysteine (L-Cys) were measured in the supernatant after reduction with dithiothreitol (DTT) and derivatisation with monobromobimane. Monobromobimane derivatives were detected fluorometrically at 480 nm by excitation at 380 nm with a Waters 474 Scanning Fluorescence Detector (Waters Co., Milford, MA, USA). Oxidised glutathione (GSSG) was detected after the irreversible alkylation of the free thiol group of GSH with NEM and the following reduction of GSSG with DTT. Reduced glutathione (GSH) concentration was then calculated by subtracting the tGSH and GSSG results. Thiol derivatives were separated on a Luna C18 100 Å column (150 × 4.6 mm, 5 mm, Phenomenex, Torrance, USA) applying a solution of 10% methanol (solvent A, pH 4.3) and 90% methanol containing 0.25% (*v*/*v*) glacial acetic acid (solvent B, pH 3.9) at a flow rate of 1 mLmin^−1^. The concentration of L-ascorbic acid (L-AA) was analysed directly in the supernatant obtained after the extraction procedure. The total concentration of ascorbate (L-AA + DHAA; DHAA—dehydroascorbic acid) was measured after complete reduction of DHAA with DTT. Separation was carried out using an AtlantisTM dC18 column (250 mm × 4.6 mm, 5 mm, Waters Co., Milford, MA, USA) coupled with a UV-VIS detector (Waters 2487) at 268 nm under isocratic conditions. The mobile phase contained 10% methanol and 2% NH_4_H_2_PO_4_ with a pH of 2.8. The concentrations of the above-mentioned compounds were calculated using a standard curve and expressed in mg*kg^−1^ FW.

Total phenolics content (TPC). TPC in leaf samples was measured with the Folin–Ciocalteu method [64] using a two-step extraction of 250 mg plant tissue with methanol in an ultrasonic bath [65]. TPC was expressed as gallic acid equivalents in mg*kg^−1^ FW.

Trolox equivalent antioxidant capacity (TEAC). Three different well-documented tests were applied to evaluate TEAC in leaves: FRAP [29,66], ABTS [67] and DPPH assays [68]. The results were then calculated using a calibration curve and expressed as mmol TE kg^−1^ FW (TE—trolox equivalents).

Antioxidative enzyme activity. Leaf tissue powder was weighed (200 mg) and suspended in 100 mM potassium phosphate buffer (5 mL, pH 7.8) containing insoluble polyvinylpolypyrrolidone (PVPP), Triton X–100 (0.5%) and ascorbate (5 mM). The mixture was centrifuged at 48,000 *g* for 20 min at 4 °C. The activity of APX (ascorbate peroxidase, EC 1.11.1.11), CAT (catalase, EC 1.11.1.6) and GR (glutathione reductase, EC 1.6.4.2) was measured spectrophotometrically (HITACHI UV-Vis spectrophotometer, model U2900, Kyoto, Japan) by monitoring the decrease in absorbance at 290 nm, 240 nm and 340 nm for APX, CAT and GR, respectively [29]. The enzyme activity was expressed in nanokatals per gram of fresh weight (nkat g^−1^ FW).

### 3.4. Statistical Analysis and Presentation of Data

The data obtained were elaborated by two-way analysis of variance (ANOVA) using Statistica version 13.0 software (TIBCO Software Inc., http://statistica.io—access date 7 July 2017, USA). Due to varying weather conditions in each season and large differences in results between cultivars, each year’s results were elaborated separately for each cultivar tested. N level and leaf sampling time were the main factors. As the interaction between these factors did not in principle appear (Table 1; Table 3), the graphs and tables show the main factor effect of antioxidant concentration and antioxidant capacity.

The significance of the differences between means was evaluated using Tukey’s HSD test at a 5% probability level. A Pearson correlation analysis between antioxidant capacity (FRAP, ABTS, DPPH tests) and the tested enzymatic and non-enzymatic antioxidants was performed (Table 2).

## 4. Conclusions

Kiwiberry (*Actinidia arguta* (Siebold et Zucc.) Planch. ex Miq.) leaf enzymatic and non-enzymatic antioxidants under different N regimes were tested three times in field conditions during the growing season for two commercially important cultivars (‘Weiki’ and ‘Geneva’). Leaf total antioxidant capacity using ABTS, DPPH and FRAP tests was evaluated over three years that experienced different weather conditions. In the context of fruit plant leaf antioxidant quality, there has been little examination previously of factors other than genetic ones, such as edaphoclimatic conditions, agronomic approaches or the plant development phase, and in the case of *Actinidia arguta* leaves, there appears to be no data at all. A research stimulus was that fruit leaves could be used as an extraordinary source of value-added compounds. The ascorbate–glutathione cycle and related enzymes (GR, APX, CAT) showed considerable changes in concentration/activity, indicating a close relationship with the plant development cycle and high sensitivity to overall external conditions. Leaf global phenolic variation was rather low throughout the growing season and was predominantly cultivar dependent. The strength of the correlation between individual antioxidants and TEAC at specific time points reflected antioxidant dynamics during the growing season. For most components tested, the close positive relationship in the case of ABTS and FRAP tests, but not DPPH, was revealed. Leaf L-AA, global phenolics, APX, CAT and TEAC also depended on the N level, while thiol compounds were not affected. Over the three years, TEAC decreased with increasing soil N fertility, and the intensity of the N effect was year dependent. These results suggest that the antioxidant properties of kiwiberry leaves are closely related to the plant development phase, which with proper N handling might be a suitable way for the acquisition of plant material with greater biological activity and potential health benefits.

## Figures and Tables

**Table 1:** Summary of analysis of variance (ANOVA) for the components tested. Values of F for particular sources of variation and their significance depending on the cultivar.

	Cultivar											
	‘Weiki’						‘Geneva’					
Component	Source of Variation											
	Time (A)		N Level (B)		Interaction:		Time (A)		N Level (B)		Interaction:	
					AB						AB	
df	2		2		4		2		2		4	
L-AA	95.4	***	16.1	***	1.26	ns	151	***	18.9	***	5.92	**
L-AA + DHAA	103	***	2.15	ns	2.74	ns	68.4	***	0.25	ns	0.46	ns
L-Cys	6.82	**	0.92	ns	1.86	ns	66.9	***	0.91	ns	0.82	ns
GSH	35.2	***	0.35	ns	1.99	ns	62.6	***	0.26	ns	1.17	ns
GSH + GSSG	44.1	***	0.34	ns	2.19	ns	60	***	0.21	ns	1.17	ns
TPC	2.58	ns	3.22	ns	1.69	ns	15.2	***	16	***	4.49	*
GR	23.6	***	0.58	ns	0.37	ns	8.67	**	0.11	ns	1.54	ns
APX	5.78	*	18.9	***	7.01	**	96.3	***	7.3	**	3.85	*
CAT	40.8	***	6.59	**	4.57	*	11.7	***	9.71	**	3.47	*

*** significant at α = 0.001; ** significant at α = 0.01; * significant at α = 0.05; ns—non-significant; L-AA: reduced ascorbate; L-AA + DHAA: total ascorbate; L-CYS: L-cysteine; GSH: reduced glutathione; GSH + GSSG: total glutathione; TPC: total phenolics; GR: glutathione reductase; APX: ascorbate peroxidase; CAT: catalase.

**Table 2:** Correlation coefficient between the individual antioxidant tested and total antioxidant capacity (TEAC) during the 2015 growing season made across two cultivars.

TEAC		Component								
	L-AA	L-AA + DHAA	L-CYS	GSH	GSH + GSSG	TPC	GR	APX	CAT	N Leaf Content
		Time								
		T1								
ABTS	0.56	0.26	0.61	0.46	0.40	0.52	−0.10	−0.65	0.22	−0.50
DPPH	−0.40	−0.18	−0.40	−0.50	−0.50	−0.50	0.26	0.48	−0.21	0.34
FRAP	0.51	0.26	0.69	0.56	0.50	0.65	−0.32	−0.81	0.42	−0.71
		T2								
ABTS	0.60	0.72	0.21	0.02	0.04	0.59	−0.05	0.73	0.73	−0.71
DPPH	−0.57	−0.72	−0.12	0.11	−0.01	−0.56	−0.02	−0.61	−0.59	0.60
FRAP	0.59	0.74	0.26	0.09	0.11	0.66	0.01	0.73	0.77	−0.76
		T3								
ABTS	0.06	0.35	0.79	0.75	0.75	0.53	0.48	0.21	0.64	−0.64
DPPH	−0.04	−0.47	−0.49	−0.46	−0.46	−0.53	−0.36	−0.20	−0.51	0.37
FRAP	0.00	0.17	0.67	0.65	0.65	0.41	0.39	0.09	0.59	−0.64

ABTS: 2,2′-azino-bis(3-ethylbenzothiazoline-6-sulphonic acid); DPPH: 2,2-diphenyl-1-picrylhydrazyl; FRAP: ferric reducing antioxidant power; L-AA: reduced ascorbate; L-AA + DHAA: total ascorbate; L-CYS: L-cysteine; GSH: reduced glutathione; GSH + GSSG: total glutathione; TPC: total phenolics; GR: glutathione reductase; APX: ascorbate peroxidase; CAT: catalase; T1—(28.05.15) the stage of fully developed leaves, T2—(16.07.15) the peak of the most intensive fruit growth and T3—(27.08.15) the beginning of fruit physiological maturity.

**Table 3:** Summary of analysis of variance (ANOVA) for antioxidant capacity tests. Values of F for particular sources of variation and their significance depending on the cultivar.

	Cultivar											
	‘Weiki’						‘Geneva’					
Component	Source of Variation											
	Sampling Time (A)		N Level		Interaction:		Sampling Time (A)		N Level		Interaction:	
			(B)		AB				(B)		AB	
df	2		2		4		2		2		4	
Antioxidant capacity test												
	ABTS											
2015	17.9	***	4.78	*	0.35	ns	10.8	***	3.80	*	0.82	ns
2016	3.65	*	1.88	ns	0.78	ns	2.92	ns	7.89	**	0.56	ns
2017	10.5	***	3.50	ns	2.48	ns	11.8	***	6.25	**	2.15	ns
	DPPH											
2015	5.36	*	0.46	ns	0.84	ns	5.34	*	1.98	ns	0.46	ns
2016	1.86	ns	2.38	ns	0.41	ns	30.0	***	5.87	*	2.13	ns
2017	0.21	ns	6.78	**	1.23	ns	0.21	ns	1.25	ns	4.88	**
	FRAP											
2015	20.2	***	5.31	*	0.96	ns	11.8	***	4.42	*	0.90	ns
2016	17.5	***	6.10	**	1.23	ns	11.5	***	7.25	**	1.22	ns
2017	6.13	**	6.21	**	0.46	ns	16.1	***	10.5	***	4.72	**

*** significant at α = 0.001; ** significant at α = 0.01; * significant at α = 0.05; ns—non-significant; ABTS: 2,2′-azino-bis(3-ethylbenzothiazoline-6-sulphonic acid); DPPH: 2,2-diphenyl-1-picrylhydrazyl; FRAP: ferric reducing antioxidant power.

**Table 4:** Concentration of different bioactive compounds (mg kg^−1^ FW) in *A. arguta* leaves depending on cultivar and soil N level.

	N Level		
Cultivar	N1	N2	N3
Component			
Reduced ascorbate (L-AA)			
‘Weiki’	1175 ± 741 b	957 ± 660 b	608 ± 490 a
‘Geneva’	1334 ± 823 b	864 ± 790 a	807 ± 638 a
Total ascorbate (L-AA + DHAA)			
‘Weiki’	1644 ± 592 a	1891 ± 887 a	1703 ± 958 a
‘Geneva’	2196 ± 1009 a	2139 ± 997 a	2068 ± 962 a
L-Cysteine (L-Cys)			
‘Weiki’	1.28 ± 0.22 a	1.47 ± 0.25 a	1.41 ± 0.54 a
‘Geneva’	2.22 ± 0.72 a	2.39 ± 0.72 a	2.34 ± 0.74 a
Reduced glutathione (GSH)			
‘Weiki’	239 ± 72 a	253 ± 82 a	234 ± 129 a
‘Geneva’	297 ± 100 a	311 ± 100 a	305 ± 103 a
Total glutathione (GSH + GSSG)			
‘Weiki’	249 ± 79 a	263 ± 88 a	246 ± 132 a
‘Geneva’	304 ± 98 a	317 ± 98 a	310 ± 102 a
Total phenolics (TPC)			
‘Weiki’	4571 ± 1214 a	3792 ± 757 a	3633 ± 765 a
‘Geneva’	6408 ± 1820 c	5396 ± 1328 b	4210 ± 750 a

Presented data are means for the three sampling times and three replications (n = 9 ± SD) obtained in 2015. Values marked with a different letter (a, b, c) in rows differ significantly at *p* ≤ 0.05 (Tukey test). N1, N2, N3—30, 50 and 80 mg N kg^−1^ DW respectively; DHAA: dehydroascorbic acid; GSSG: oxidised glutathione.

**Table 5:** Activity of antioxidative enzymes (nkat g^−1^ FW) in *A. arguta* leaves depending on cultivar and soil N level.

	N Level		
Cultivar	N1	N2	N3
Enzyme			
Glutathione reductase (GR)			
‘Weiki’	16.0 ± 3.4 a	15.3 ± 4.6 a	16.7 ± 5.8 a
‘Geneva’	17.7 ± 2.7 a	17.2 ± 4.2 a	17.1 ± 3.9 a
Ascorbate peroxidase (APX)			
‘Weiki’	110 ± 47 a	184 ± 41 b	108 ± 50 a
‘Geneva’	178 ± 105 b	132 ± 62 a	135 ± 91 a
Catalase (CAT)			
‘Weiki’	5.26 ± 3.9 a	7.49 ± 2.9 b	5.86 ± 1.77 a
‘Geneva’	13.0 ± 2.57 b	10.4 ± 1.38 a	10.5 ± 2.21 a

Presented data are means for three sampling times and three replications (n = 9 ± SD) obtained in 2015. Values marked with a different letter (a, b) in rows differ significantly at *p* ≤ 0.05 (Tukey test). N1, N2, N3—30, 50 and 80 mg N kg^−1^ DW respectively.

**Table 6:** Total antioxidant capacity in ABTS, DDPH and FRAP assay tests (mmol TE kg^−1^ FW) in two *A. arguta* cultivars’ leaves depending on soil N level in different growing seasons.

	Cultivar					
	‘Weiki’			‘Geneva’		
	N Level					
Year	N1	N2	N3	N1	N2	N3
Antioxidant capacity test						
ABTS						
2015	245 ± 22 b	222 ± 37 ab	218 ± 31 a	309 ± 44 b	291 ± 48 ab	265 ± 45 a
2016	252 ± 55 a	233 ± 41 a	212 ± 42 a	285 ± 37 b	238 ± 29 a	237 ± 24 a
2017	185 ± 28 b	149 ± 44 a	161 ± 52 ab	250 ± 64 b	201 ± 41 a	192 ± 56 a
DPPH						
2015	320 ± 30 a	332 ± 39 a	336 ± 53 a	268 ± 47 a	303 ± 44 a	296 ± 46 a
2016	220 ± 27 a	202 ± 21 a	194 ± 28 a	252 ± 38 b	213 ± 56 a	224 ± 46 ab
2017	198 ± 43 b	150 ± 18 a	153 ± 24 a	197 ± 37 a	184 ± 43 a	175 ± 29 a
FRAP						
2015	149 ± 21 b	129 ± 25 a	127 ± 30 a	203 ± 34 b	183 ± 37 ab	169 ± 30 a
2016	112 ± 17 b	103 ± 18 ab	92 ± 23 a	134 ± 18 b	114 ± 23 a	112 ± 15 a
2017	93 ± 17 b	76 ± 21 ab	68 ± 15 a	130 ± 43 b	97 ± 22 a	96 ± 23 a

Presented data are means for three sampling times and three replications (n = 9 ± SD) obtained in 2015, 2016 and 2017. Values in rows marked with a different letter (a, b, ab) differ significantly at *p* ≤ 0.05 (Tukey test). Data were analysed separately for each year and each cultivar; N1, N2, N3—30, 50 and 80 mg N kg^−1^ DW respectively; ABTS: 2,2′-azino-bis(3-ethylbenzothiazoline-6-sulphonic acid); DPPH: 2,2-diphenyl-1-picrylhydrazyl; FRAP: ferric reducing antioxidant power; TE: trolox equivalents.

## Data Availability

Not applicable.

## Sample Availability

Samples of the compounds are not available from the authors.
